# CD204-positive M2-like tumor-associated macrophages increase migration of gastric cancer cells by upregulating miR-210 to reduce NTN4 expression

**DOI:** 10.1007/s00262-023-03601-5

**Published:** 2024-01-04

**Authors:** Chin-Wang Chen, Hao-Chen Wang, I-Min Tsai, I-Shu Chen, Chang-Jung Chen, Ya-Chin Hou, Yan-Shen Shan

**Affiliations:** 1 Department of Surgery, Kaohsiung Veterans General Hospital Tainan Branch, Tainan, Taiwan; 2 Institute of Clinical Medicine, College of Medicine, National Cheng Kung University, Tainan, Taiwan; 3 Medical Imaging Center, Innovation Headquarters, National Cheng Kung University, Tainan, Taiwan; 4 Department of Surgery, Kaohsiung Veterans General Hospital, Kaohsiung, Taiwan; 5 Department of Surgery, National Cheng Kung University Hospital, College of Medicine, National Cheng Kung University, 138, Sheng-Li Road, Tainan, 70428 Taiwan

**Keywords:** Tumor microenvironment, Tumor-associated macrophage, Migration, miR-210, NTN4, Gastric cancer

## Abstract

**Background:**

Tumor-associated macrophages (TAMs) are the predominant immune cells in the tumor microenvironment and portend poor prognosis. However, the molecular mechanisms underlying the tumor promotion of TAMs have not been fully elucidated.

**Methods:**

Coculture of gastric cancer cells with U937 cells was performed to investigate the impact of TAMs on cancer cell behavior. MicroRNA (miRNA) microarray and bioinformatics were applied to identify the involved miRNAs and the functional target genes. The regulation of the miRNA on its target gene was studied using anti-miRNA and miRNA mimic.

**Results:**

Coculture with CD204^+^ M2-like TAMs increased proliferation, migration, and epithelial-mesenchymal transition of gastric cancer cells. MiR-210 was the most upregulated miRNA in cancer cells identified by miRNA microarray after coculture. In gastric cancer tissues, miR-210 expression was positively correlated with CD204^+^ M2-like TAM infiltration. Inactivation of miR-210 by antimir attenuated CD204^+^ M2-like TAMs-induced cancer cell migration. Using pharmacological inhibitors and neutralizing antibodies, CD204^+^ M2-like TAMs-secreted TNFα was found to upregulate miR-210 through NF-κB/HIF-1α signaling. Bioinformatics analysis showed netrin-4 (NTN4) as a potential target of miR-210 to suppress gastric cancer cell migration. We also found an inverse expression between miR-210 and NTN4 in cancer cells after coculture or in tumor xenografts. Anti-miR-210 increased NTN4 expression, while miR-210 mimics downregulated NTN4 in cancer cells. Reporter luciferase assays showed that MiR-210 mimics suppressed *NTN4* 3’ untranslated region-driven luciferase activity in cancer cells, but this effect was blocked after mutating miR-210 binding site.

**Conclusions:**

CD204^+^ M2-like TAMs can utilize the TNF-α/NF-κB/HIF-1α/miR-210/NTN4 pathway to facilitate gastric cancer progression.

**Supplementary Information:**

The online version contains supplementary material available at 10.1007/s00262-023-03601-5.

## Introduction

Chronic inflammatory insults are currently believed to be a precursor of malignancy development and correlates with cancer progression [1]. Gastric cancer, one of the most common cancers worldwide, arises in close association with chronic inflammation [2]. Atrophic gastritis, persistent chronic inflammation of the gastric mucosa, reportedly has a 5.7-fold increased risk for developing gastric cancer [3, 4]. Tumor-associated macrophages (TAMs) are a major component of the inflammatory infiltrate in the tumor microenvironment (TME), which has been considered a key player in connecting inflammation and cancer [5]. TAM infiltration has been widely accepted as a negative prognostic parameter for most solid tumors [6].

TAMs can be classified into M1 and M2 phenotypes. M1 TAMs, characterized by the production of IL-12, IL-23, and inducible nitric oxide synthase (iNOS), can be activated by IFN-γ, lipopolysaccharides (LPS), and TNF-α to exert cytotoxic effects against cancer [7]. On the other hand, M2 TAMs, arising from exposure to T helper type 2 (Th2) cytokines, exhibit protumor properties and express high levels of IL-10 and scavenger receptors [7]. Clinicopathological studies have shown that the infiltration of M2 TAM in gastric cancer was associated with more malignant phenotypes, such as higher microvascular density, increased depth of invasion, more lymph node involvement, and advanced tumor stages [8, 9]. A high M2 TAM content in solid tumors rendered the prognosis dismal [10]. However, despite increasing evidence for a protumor role of TAMs, the way in which these cells contribute to cancer aggressiveness still needs full elucidation.

MicroRNAs (miRNAs) are a class of short and noncoding endogenous RNA molecules that are able to hinder gene expression at both the transcription and translation levels by binding to the 3’ untranslated region (3’ UTR) of the target genes [11]. Since miRNAs regulate up to 30% of all human genes, a single miRNA may govern expression of multiple target genes or pathways involving in complex signaling networks [12]. Therefore, dysregulation of miRNAs may contribute to the pathogenesis of many human disorders including cancer. Recent studies have highlighted the important role of miRNAs in cancer that nearly 50% of known miRNA genes are located in cancer-associated genomic regions and frequently deleted or amplified in tumorigenesis [13]. Aberrant expression of miRNAs in human malignancies has also been documented, [14, 15] suggesting their potential as diagnostic and prognostic markers and therapeutic targets. Recently, inflammation has been implicated in the regulation of miRNAs. For instance, STAT3, activated by IL-6, has been demonstrated to activate miR-21 and miR-181b-1 to target PTEN and CYLD, respectively, consequently resulting in cell transformation [16]. The tumor suppressor miR-7 was down-regulated in gastritis and gastric tumors in response to activated macrophages-derived small molecules [17]. Accordingly, miRNAs may act as mediators of inflammation-driven carcinogenesis. In this study, the miRNA microarray data showed that TAMs induced miR-210 expression in gastric cancer cells; therefore, we were interested in studying the role of miR-210 in the interaction between TAMs and gastric cancer cells.

## Results

### CD204^+^ M2-like TAMs enhance the malignant behavior of gastric cancer cells and confer poor patient survival

**Fig. 1:**
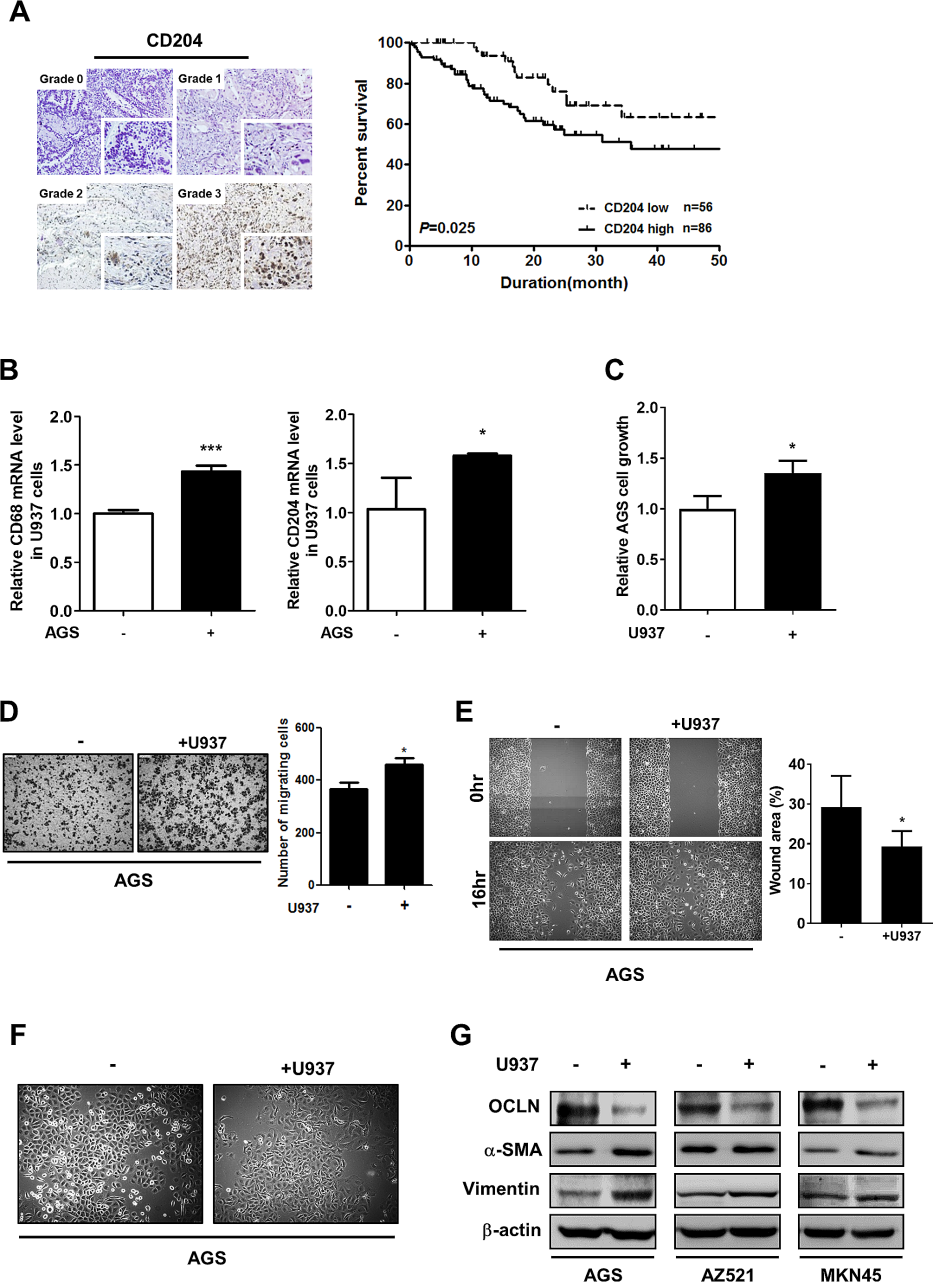
CD204^+^ M2-like TAMs increase cancer cell migration and miR-210 expression. **(A)** Representative images show CD204^+^ cells in human gastric tumor specimens (left). Staining positivity was graded as 0 (no cells with positive staining), 1 (less than 25% cells with positive staining), 2 (25–50% cells with positive staining), and 3 (more than 50% cells with positive staining). Kaplan-Meier analysis was performed to determine the association between CD204 expression and the overall survival in 142 gastric cancer patients. **(B)** U937 cells were monocultured or cocultured with AGS cells for 72 h. Cell lysates of U937 cells were subjected to Western blot analysis of CD68 and CD204. **(C)** Seventy-two hours after coculture with U937 cells, AGS cell growth was measured using the cell counters. ∗ *P* < 0.05 versus monoculture control, Student’s t-test. **(D)** Seventy-two hours after coculture with U937 cells, the migration of AGS cells was analyzed by the Transwell migration assay (left). The bar graph indicates the numbers of migrating cells (right). ∗ *P* < 0.05 versus monoculture control, Student’s t-test. **(E)** After 72 h of coculture with U937 cells, AGS cells were collected and grown in 2-well culture-insert to generate wounds. The wound area was measured from 0 to 16 h. The bar graph indicates the percentages of wound areas (right). ∗ *P* < 0.05 versus monoculture control, Student’s t-test. **(F)** Morphology of AGS cells after 72 h of U937 coculture was observed with an optical microscope. **(G)** Expression of EMT markers in cancer cells cocultured with or without U937 cells for 72 h was measured by Western blotting

High M2 TAM infiltration frequently occurs in gastric cancer tissues and is strongly associated with poor patient survival [18, 19]. IHC staining for the specific M2 marker CD204 in 142 gastric cancer specimens was performed, and the staining was scored as grade 0 (no cells with positive staining), grade 1 (less than 25% cells with positive staining), grade 2 (25–50% cells with positive staining), and grade 3 (more than 50% cells with positive staining). Kaplan-Meier survival analysis showed that patients with high CD204 expression (CD204 staining positivity > 50%) had worse overall survival than those with low CD204 expression (Fig. 1A). In order to study the interaction between inflammatory cells and cancer cells, the in vitro macrophages-cancer cells coculture system was used to mimic the inflammatory microenvironment. U937 cells are able to differentiate into M2 TAMs in response to tumor-derived factors [20]. Coculture with AGS cells for 72 h induced the polarization of U937 cells into M2-like TAMs characterized by increased expression of the macrophage marker CD68 and the M2 marker CD204 (Fig. 1B), which in turn increased the growth (Fig. 1C) and the migration ability of AGS cells (Fig. 1D and E). A more mesenchymal-like morphology (Fig. 1F), down-regulation of the epithelial marker Occludin (Fig. 1G), and increased expression of the mesenchymal markers α-SMA and Vimentin (Fig. 1G) were also seen in cancer cells.

### MiR-210 contributes to CD204^+^ M2-like TAMs-induced gastric cancer cell migration

**Fig. 2:**
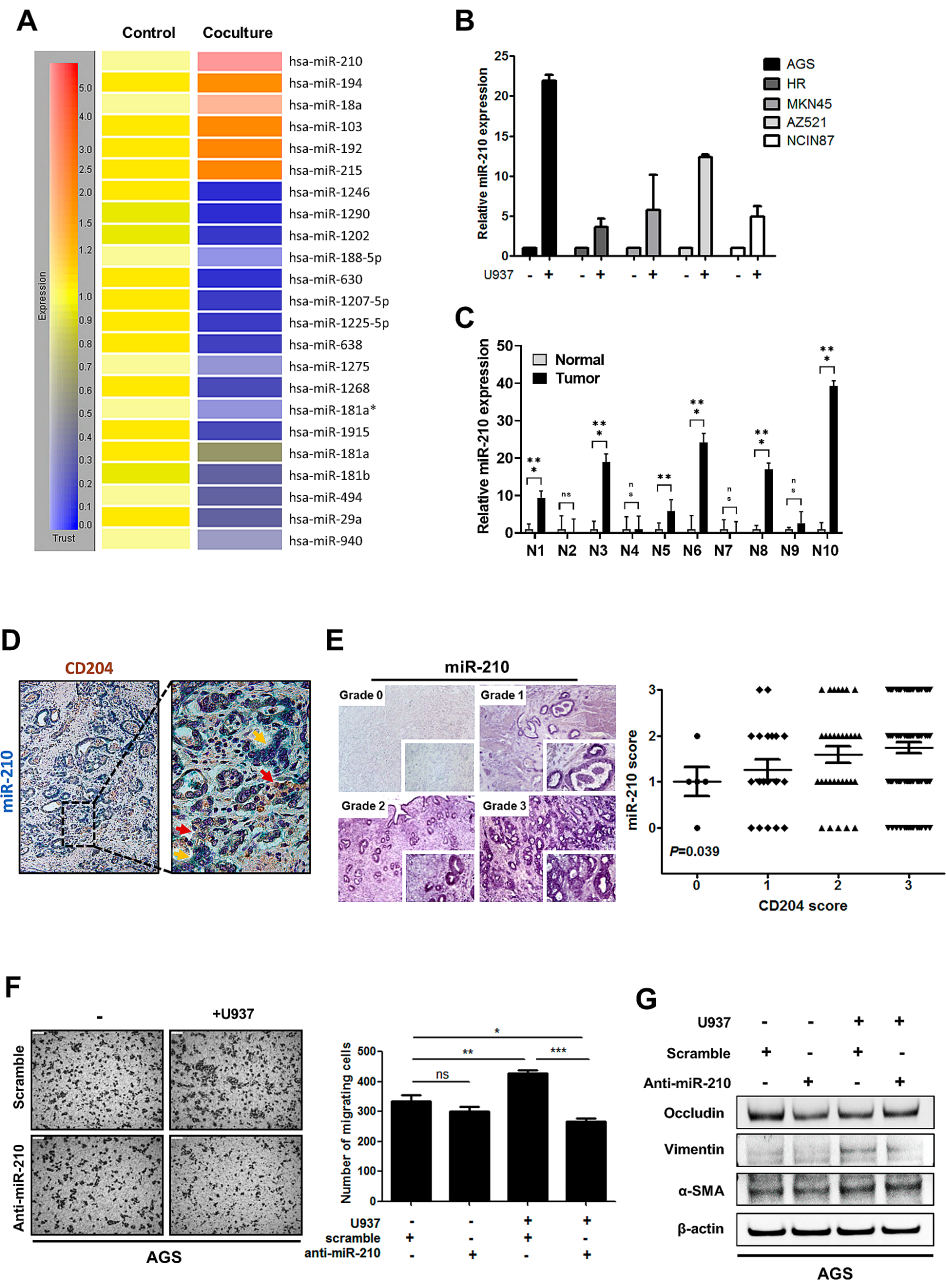
CD204^+^ M2-like TAMs-induced cancer cell migration is mediated through miR-210. **(A)** MiRNA expression microarray analysis shows the top 20 downregulated and upregulated miRNAs in AGS cells cocultured with U937 cells in comparison with AGS monocultures. **(B)** Expression of miR-210 in gastric cancer cells cocultured with or without U937 cells was determined by qPCR. The bar graph depicts the relative miR-210 expression. **(C)** Expression of miR-210 in gastric tumor tissues and their matched adjacent noncancerous tissues were determined by qPCR. The bar graph depicts the relative miR-210 expression. **(D)** Representative dual IHC-ISH staining for CD204 (brown) and miR-210 (indigo) in human gastric tumor tissues. Magnification: 100X (left). The image (right) is high-magnification (400X) of the areas outlined by black squares. Red arrows indicate M2 TAMs. Yellow arrows indicate cancer cells expressing miR-210. **(E)** Representative images show miR-210-positive cells in human gastric tumor specimens (left). Staining positivity was graded as 0 (no cells with positive staining), 1 (less than 25% cells with positive staining), 2 (25–50% cells with positive staining), and 3 (more than 50% cells with positive staining). The correlation between CD204 and miR-210 expression was evaluated using Kendall’s tau correlation (Kendall’s tau-c = 0.151, *P* = 0.039; right). **(F)** AGS cells were treated with or without miR-210-specific hairpin inhibitor for 24 h prior to coculture with U937 cells for 72 h. The migration of AGS cells was determined by the Transwell migration assay (left). The bar graph indicates the numbers of migrating cells (right). NS, not significant; ∗ *P* < 0.05; ∗∗ *P* < 0.01; ∗∗∗ *P* < 0.001, significant differences between groups, one-way ANOVA. **(G)** Expression of EMT markers in AGS cells that were treated with or without miR-210-specific hairpin inhibitor for 24 h prior to coculture with U937 cells for 72 h was measured by Western blotting

To identify the factors involved in CD204^+^ M2-like TAMs-induced cancer cell migration, microarray-based miRNA profiling analysis in AGS cells after coculture was conducted. Among the top differentially expressed miRNAs, miR-210 was the most upregulated one with 12.8-fold difference compared to the control group (Fig. 2A). We further validated the microarray data by performing quantitative real-time PCR in human gastric cancer cell lines and tumor tissues. Expression of miR-210 in cancer cell lines after coculture was higher than those in monocultures (Fig. 2B). Similar results could also be seen in AGS cells after coculture with cytokine-induced M2-like macrophages from U937 cells, monocytic THP-1 cells, or monocyte-derived macrophages (Supplementary Figure S1). Elevated expression of miR-210 was more frequently detected in gastric cancer tissues than in the adjacent normal gastric tissues (Fig. 2C). Furthermore, the correlation between miR-210 expression and CD204^+^ M2-like TAM density was verified in 142 human gastric tumors. MiR-210 expression and CD204 expression were detected by ISH and IHC staining, respectively. The result show that miR210-expressing tumor cells were surrounded by CD204^+^ M2-like TAMs in close juxtaposition (Fig. 2D). After the staining was scored into 4 grades, the scatter plot of scoring revealed a positive correlation between miR-210 and CD204 expression (*P* = 0.039; Fig. 2E), suggesting that miR-210 expression in gastric cancer cells could be regulated by CD204^+^ M2-like TAM TAMs. Because miR-210 expression has been associated with cell migration and metastasis in several types of cancers, [21–26] we sought to ascertain whether miR-210 mediates CD204^+^ M2-like TAMs-induced cancer cell migration. After coculture, the migration ability of AGS cells was increased, and inactivation of miR-210 by the miR-210-specific hairpin inhibitor effectively counteracted the promotion (Fig. 2F), demonstrating that CD204^+^ M2-like TAMs promote cancer cell migration by upregulating miR-210.

### CD204^+^ M2-like TAMs induce miR-210 expression via the TNF-α/NF-κB/HIF-1α pathway

**Fig. 3:**
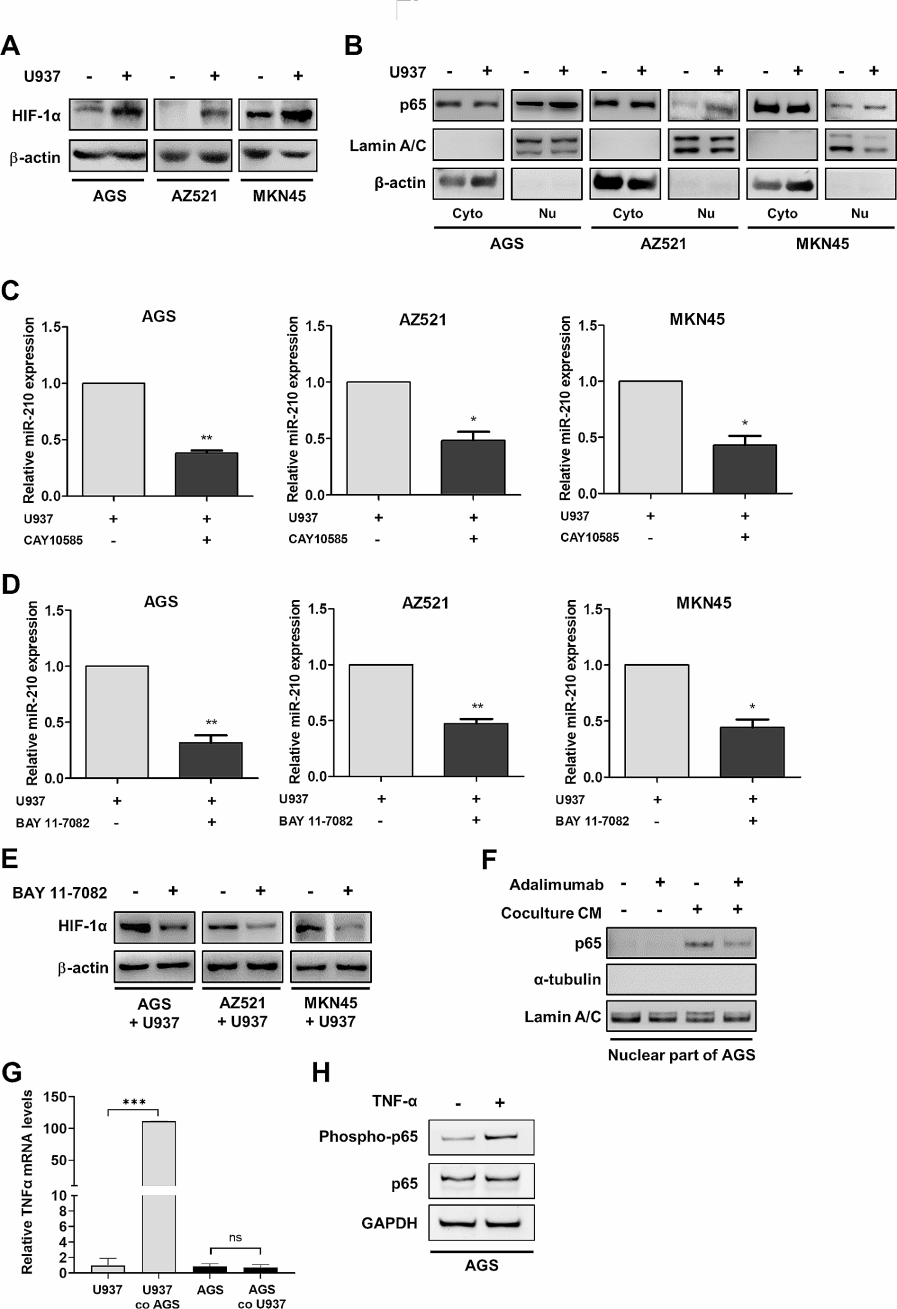
CD204^+^ M2-like TAMs upregulate miR-210 in cancer cells by activating NF-κB and HIF-1α via TNF-α. **(A)** Cancer cells were cocultured with U937 cells for 72 h. The cancer cell lysates were subjected to Western blotting to detect HIF-1α expression. **(B)** Seventy-two hours after coculture with U937 cells, cancer cells were lysed and fractionated. Cytoplasmic (Cyto) and nuclear (Nu) extracts were subjected to Western blot analysis for NF-κB p65 detection. Lamin A/C and β-actin were used as a nuclear marker/control and a cytoplasmic marker/control, respectively. **(C)** After U937 coculture, cancer cells were treated with CAY10585 (10 µM) to inhibit HIF-1α. Expression of miR-210 in cancer cells was assessed by qPCR. The bar graphs depict the relative expression of miR-210. ∗ *P* < 0.05; ∗∗ *P* < 0.01 versus control, Student’s t-test. **(D-E)** After U937 coculture, cancer cells were treated with the NF-κB inhibitor BAY 11-7082 (5 µM). Expression of miR-210 in cancer cells was assessed by qPCR, and HIF-1α protein expression in cancer cells was measured by Western blot analysis. The bar graphs depict the relative expression of miR-210. ∗ *P* < 0.05; ∗∗ *P* < 0.01 versus control, Student’s t-test. **(F)** AGS cells were treated with conditioned medium (CM) from AGS/U937 coculture in the presence or absence of the TNF-α neutralizing antibody adalimumab (10 µg/mL) for 3 h. Nuclear (Nu) extracts of AGS cells were subjected to Western blot analysis for NF-κB p65 detection. The nuclear marker Lamin A/C was used as an internal control. **(G)** AGS cells and U937 cells were monocultured or cocultured for 72 h. Expression of TNF-α mRNA in AGS cells and U937 cells after monoculture or coculture was measured by qPCR. The bar graphs depict the relative expression of TNF-α mRNA. ∗∗∗ *P* < 0.001 versus monoculture control, Student’s t-test. **(H)** AGS cells were treated with TNF-α recombinant protein (10 ng/mL) for 3 h, and expression of phosphorylated p65 and total p65 protein was detected by Western blotting

MiR-210 has been demonstrated to be the most consistently and robustly induced miRNA under hypoxia in different types of tumor cells and normal cells [27]. The transcription factor HIF-1 is a master regulator in controlling the adaptive response to hypoxia in cells. Besides hypoxia, HIF-1 protein is also upregulated by the inflammatory mediator NF-κB to promote tumor growth and metastasis, suggesting that HIF-1 links inflammation to oncogenesis [28]. We therefore sought to determine whether the upregulation of miR-210 induced by CD204^+^ M2-like TAMs is mediated by NF-κB and HIF-1α in cancer cells. Co-culture with U937 cell indeed increased HIF-1α expression (Fig. 3A) and enhanced nuclear translocation of NF-κB (Fig. 3B) in AGS, AZ521, and MKN45 cells. Inhibition of HIF-1α using CAY10585 reduced miR-210 expression (Fig. 3C). Treatment with BAY 11-7082 to suppress NF-κB activity downregulated not only miR-210 (Fig. 3D) but also HIF-1α (Fig. 3E), suggesting NF-κB is an upstream regulator of HIF-1α. Furthermore, neutralizing TNF-α with adalimumab could reduce coculture-induced nuclear translocation of NF-κB (Fig. 3F), suggesting the involvement of TNF-α in the interaction between gastric cancer cells and CD204^+^ M2-like TAMs. We also identified the cell source of TNF-α in the coculture, and found that after 72 h of coculture, expression of TNF-α mRNA was not changed in AGS cells compared with AGS monoculture. However, expression of TNF-α mRNA was greatly upregulated in U937 cells after coculture with AGS cells for 72 h compared with U937 monoculture (Fig. 3G), suggesting that CD204^+^ M2-like TAMs were the major source of TNF-α release. In addition, we added exogenous TNF-α to AGS cells and found that p65 could be indeed activated (Fig. 3H). Taken together, these results demonstrate that the TNFα/NF-κB/HIF-1α pathway may contribute to CD204^+^ M2-like TAMs-dependent induction of miR-210 expression in gastric cancer cells.

### MiR-210 expression is inversely correlated with NTN4 expression

**Fig. 4:**
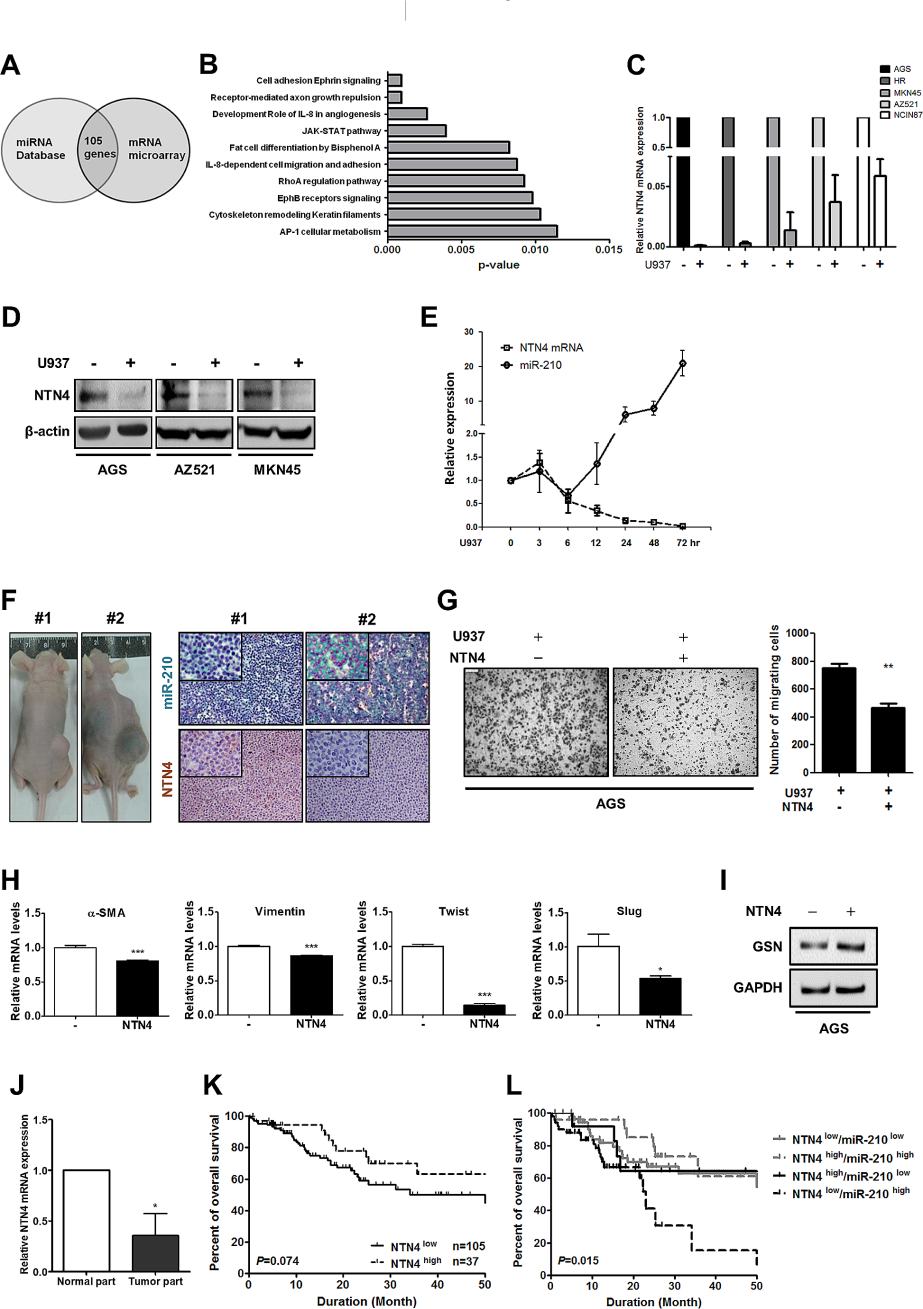
NTN4 expression is repressed by CD204^+^ M2-like TAMs and has a negative correlation with miR-210. **(A)** Schema of the candidate genes identified by two independent approaches. The left circle represents a bioinformatics prediction of putative target genes of miR-210. The right circle indicates the genes downregulated in AGS-U937 coculture, which were identified by mRNA microarray analysis. Through the intersection of these two gene sets, 105 genes were obtained. **(B)** Metacore analysis of putative target genes. **(C)** After monoculture and U937 coculture, mRNA expression of NTN4 in cancer cells was determined by qPCR. The bar graph depicts the relative *NTN4* mRNA expression. **(D)** After monoculture and U937 coculture, protein expression of NTN4 in cancer cells was analyzed by Western blotting. **(E)** AGS cells were cocultured with U937 cells for the indicated time points. *NTN4* mRNA and miR-210 levels were measured by qPCR, and their expression relative to the time point of 0 h was represented using a line graph. **(F)** Expression of miR-210 and NTN4 protein was detected by ISH and IHC, respectively, in AGS xenografts in mice with ascites (#2) or without ascites (#1). **(G)** AGS cells were cocultured with U937 cells. After exposure to human recombinant NTN4 (100 ng/mL), the migration of AGS cells was analyzed with the Transwell migration assay (left). The bar graph indicates the numbers of migrating cells (right). ∗∗ *P* < 0.01 versus control, Student’s t-test. **(H)** After treatment with NTN4 (100 ng/mL), mRNA expression of the mesenchymal markers including α-SMA, Vimentin, Twist, and Slug in AGS cells was determined by qPCR. The bar graph depicts the relative mRNA expression. ∗ *P* < 0.05; ∗∗∗ *P* < 0.001 versus control, Student’s t-test. **(I)** After treatment with NTN4 (100 ng/mL), GSN expression in AGS cells was measured by Western blotting. **(J)** Expression of NTN4 mRNA in human gastric tumor specimens and the matched adjacent noncancerous tissues was assessed by qPCR. **(K)** Kaplan-Meier analysis was conducted to examine the association between NTN4 protein expression and the overall survival in 142 gastric cancer patients. **(L)** The Kaplan-Meier method was performed to evaluate the association of combined expression of miR-210 and NTN4 with the overall survival in 142 gastric cancer patients

To identify the gene targets of miR-210, microarray analysis was carried out to search for the downregulated genes in AGS cells after coculture compared with that in monoculture. Among the genes with altered expression, a set of 105 genes (Table S3) were predicted to be putative targets of miR-210 by using several well-known miRNA target gene prediction programs, including TargetScan, MicroCosm, miRanda, PicTar, mirBD and microRNA.org (Fig. 4A). In order to investigate the modulatory effect of miR-210 on M2 TAMs-mediated cancer progression, enrichment analysis using the MetaCore™ platform was performed to identify the biological functions and pathways related to the 105 putative target genes of miR-210. The data show that the 105 potential target genes had a strong correlation with cell migration, and of these genes, the laminin-related secreted protein NTN4 is one of the most downregulated migration-associated genes (Fig. 4B). We then determined the function of NTN4 in gastric cancer cells. Both mRNA and protein levels of NTN4 were much lower in cancer cells after coculture than in cancer cell monocultures (Fig. 4C and D). the 72-hour time course experiment showed that there existed a negative correlation between miR-210 and NTN4 expression during coculture (Fig. 4E). The inverse correlation could be also observed in vivo. After the cell mixture of AGS cells and U937 cells was inoculated into mice to form tumors, stronger miR-210 expression and weaker NTN4 expression were found in the mice with bigger tumors and poor prognosis (#2) than in the mice with small tumors (#1) (Fig. 4F). When we used another gastric cancer cell line SCM-1 in animal experiments, similar results could also be observed (Supplementary Figure S2). We further verified function of NTN4 in gastric cancer cells and found that treatment with human recombinant NTN4 inhibited coculture-induced migration of AGS cells (Fig. 4G). Gene expression of the mesenchymal markers α-SMA, Vimentin, Twist, and Slug could be decreased by NTN4 (Fig. 4H). Gelsolin (GSN), an actin binding protein, functions as a switch to control epithelial-mesenchymal transition (EMT) in cancer. Previous studies have shown that decreased GSN expression enhanced mesenchymal marker expression to promote cell migration, invasion, and metastasis in several types of cancers such as breast cancer, glioblastoma, and gastric cancer [29–31]. We also found that, after NTN4 addition, GSN expression was increased in AGS cells (Fig. 4I), suggesting the contribution of GSN to the effect of NTN4 on cell migration. Collectively, these results indicate that NTN4 is a negative regulator of cell migration and EMT.

### The combination of low NTN4 expression and high miR-210 expression portends poor patient survival

To determine the significance of miR-210 and NTN4 in gastric cancer, we measured their expression in tumor tissues, and carried out Kaplan–Meier to estimate overall survival. *NTN4* mRNA expression was lower in gastric tumor tissues than in noncancerous parts (Fig. 4J). After IHC staining for NTN4, we further divided patients into the NTN4^high^ group (staining positivity > 50%) and the NTN4^low^ group (staining positivity ≤ 50%). NTN4 expression was marginally associated with patient outcomes (Fig. 4K). However, patients with simultaneously high miR-210 expression and low NTN4 expression had significantly worst overall survival among all groups (Fig. 4L). Taken together, NTN4 might be a potential prognostic factor for gastric cancer.

### MiR-210 suppresses NTN4 expression by directly binding to the 3’-UTR of ***NTN4*** transcript

**Fig. 5:**
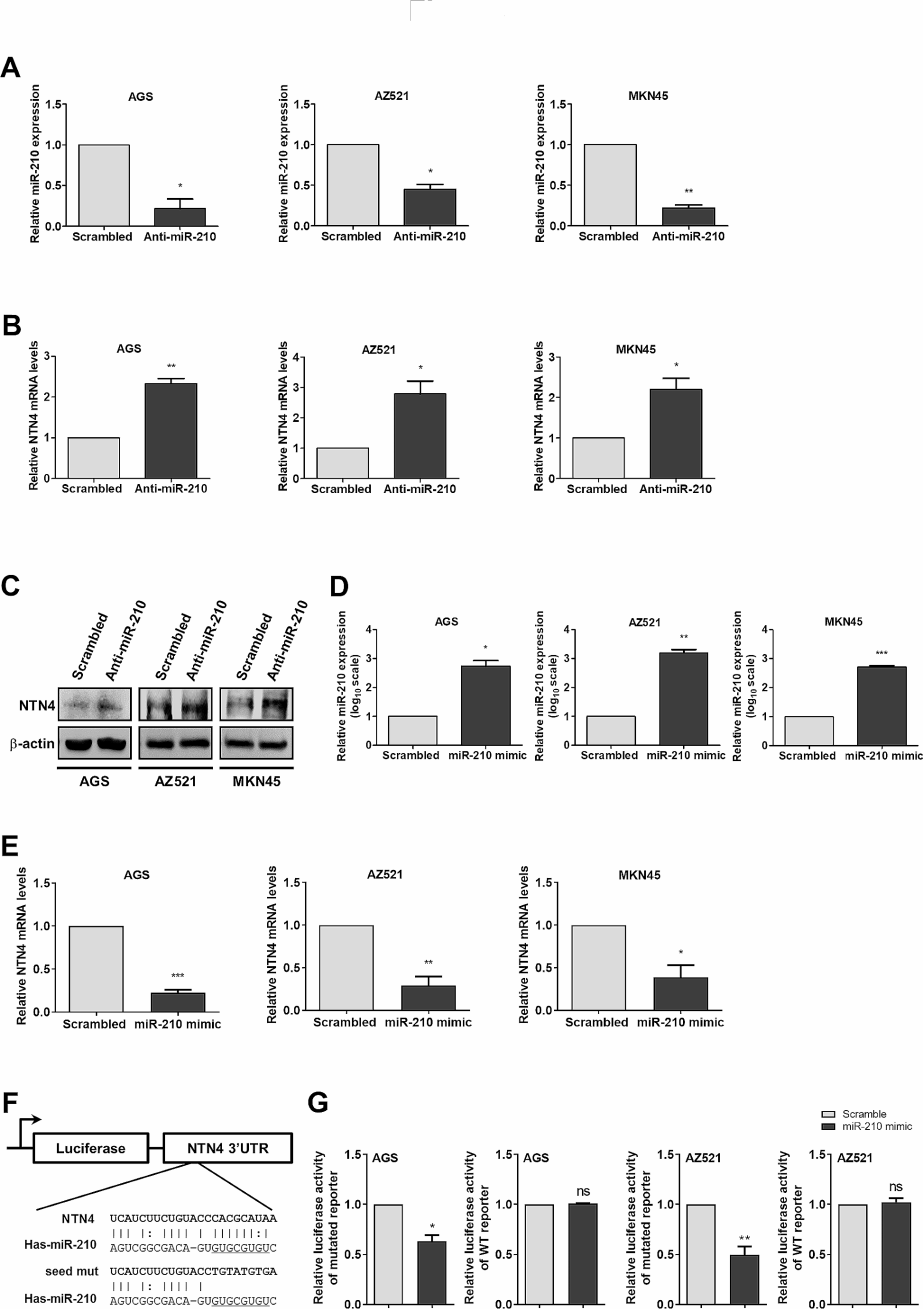
NTN4 is a direct target of miR-210. **(A)** Cancer cells were transiently transfected with miR-210 hairpin inhibitors or scrambled control for 48 hours. Expression of miR-210 was measured by qPCR. The bar graphs depict the relative expression of miR-210. ∗ *P* < 0.05; ∗∗ *P* < 0.01 versus scrambled controls, Student’s t-test. **(B-C)** Expression of *NTN4* mRNA and protein in cancer cells transfected with miR-210 hairpin inhibitors or scrambled control was measured by qPCR and Western blotting, respectively. The bar graphs depict the relative expression of *NTN4* mRNA. ∗ *P* < 0.05; ∗∗ *P* < 0.01 versus scrambled controls, Student’s t-test. **(D)** Cancer cells were transiently transfected with miR-210 mimics or scrambled control miRNAs for 48 hours. Expression of miR-210 was measured by qPCR. The bar graphs depict the relative expression of miR-210. ∗ *P* < 0.05; ∗∗*P* < 0.01; ∗∗∗ *P* < 0.001 versus scrambled controls, Student’s t-test. **(E)** Expression of *NTN4* mRNA in cancer cells transfected with miR-210 mimics or scrambled control miRNAs was determined by qPCR. The bar graphs depict the relative expression of *NTN4* mRNA. ∗ *P* < 0.05; ∗∗ *P* < 0.01 versus scrambled controls, Student’s t-test. **(F)** Construction of the wild-type and the mutant *NTN4* 3’-UTR reporters. **(G)** Luciferase activity in cancer cells cotransfected with miR-210 mimics and luciferase reporters harboring wild-type (wt) or mutant *NTN4* 3’-UTR (mut) were analyzed with the luciferase reporter assay. The bar graph depicts the relative luciferase activity. ∗ *P* < 0.05; ∗∗ *P* < 0.01 vs. scrambled controls, Student’s t-test

To verify whether NTN4 expression regulated by miR-210, we manipulated the expression of miR-210 in cancer cells by transient transfection with the miR-210 hairpin inhibitors or artificial miR-210 mimics. Suppression of miR-210 expression by the miR-210 hairpin inhibitors (Fig. 5A) increased both mRNA (Fig. 5B) and protein expression (Fig. 5C) of NTN4 in AGS, AZ521, and MKN45 cells. In contrast, transfection with synthetic miR-210 mimics to enforce expression of miR-210 (Fig. 5D) diminished *NTN4* mRNA expression in AGS, AZ521 and MKN45 cells (Fig. 5E). Furthermore, to examine whether miR-210 represses NTN4 expression by directly targeting *NTN4* mRNA transcripts, we used the MicroCosm prediction program to search for potential miR-210 binding sites on *NTN4* mRNA. We found that the 3’-UTR of *NTN4* mRNA houses a sequence that matches the 7-mer seed sequence of miR-210 (Fig. 5F). To test the prediction, the *NTN4* 3’UTR was cloned into luciferase reporter vectors. After transfection of reporter into cancer cells, we treated the cells with miR-210 mimics or non-targeting control miRNAs. The luciferase activity was reduced by approximately 40% in AGS cells and 50% in AZ521 cells in comparison with control, whereas the introduction of point mutations into the miR-210 seed sequence of the *NTN4* 3′UTR could prevent this effect (Fig. 5G). Taken together, these results clearly demonstrated NTN4 as a direct target of miR-210.

## Discussion

Highly plastic macrophages originating from circulating monocytes are capable of differentiating into TAMs in the TME to facilitate cancer progression and metastasis; [32] which has been well documented but remains a poorly understood phenomenon at the molecular level. Because both TAMs and miRNAs are highly involved in cancer-related inflammation, the correlation between TAMs and miRNA regulation in cancer cells was investigated in this study. We found that miR-210 in cancer cells was profoundly upregulated in the presence of CD204^+^ M2-like TAMs. Increased miR-210 expression promoted migration ability of cancer cells by inhibiting NTN4 expression. We further identified that the induction of miR-210 expression by CD204 + M2-like TAMs was mediated through the NF-κB/HIF-1α pathway.

In response to hypoxic stress, miR-210 can modulate many cellular adaptations such as cell proliferation, DNA repair, metabolism, cell migration, and angiogenesis [33]. Given that hypoxia commonly occurs in solid tumors, hypoxia-inducible miR-210 has also been implicated in tumor development and progression. It has been observed that miR-210 is highly expressed in several types of cancers, such as breast cancer, lung cancer, oral cancer, pancreatic cancer, and melanoma [34–38]. Increased expression of miR-210 predicts unfavorable survival in cancer patients [39]. High miR-210 expression levels are positively correlated with lymph node metastasis in pancreatic ductal adenocarcinoma patients [40]. Here, we also found that miR-210 was frequently upregulated in gastric tumors, which was associated with poor patient outcomes. In addition, CD204^+^ M2-like TAMs promoted tumor progression by regulating miR-210. Taken together, miR-210 upregulation in cancers is a common event and can be as an effective biomarker and prognostic tool in gastric cancer.

Recent research reveals that miRNAs not only exist inside cells but also detect in extracellular space. Intriguingly, miRNAs in plasma or serum remain stable under severe conditions, such as high temperatures, extreme pH values, repeated freeze-thaw cycles, and long-term storage [41–43]. The high stability of the circulating miRNAs may result from their smaller size and the protection provided by exosomes, RNA-binding proteins, or lipoprotein complexes [44–46]. Because exosomes deliver messages from donor cells to nearby or distant cells, the existence of miRNAs in exosomes suggests a role for circulating miRNAs in cell-to-cell communication. Previously, only direct contact or secretion of cytokine/chemokine is considered to mediate the communication between macrophages and tumor cells; however, increasing evidence has shown that macrophages are capable of secreting exosomes containing both genetic and protein materials to regulate tumor progression [47, 48].

NTN4 belongs to the Netrin family and acts as a regulator of neurite outgrowth and branching and cell migration [49]. Recently, increasing evidence has suggested that NTN4 plays a suppressor role in cancer. In breast cancer, only about 40% of tumors expressed detectable amounts of NTN4 at the protein level, and the expression of NTN4 was associated with better overall survival and disease-free survival [50]. Overexpression of NTN-4 impaired tumor growth and angiogenesis in a mouse xenograft model of colon cancer. Similarly, we also found lower *NTN4* mRNA expression in gastric tumor tissues compared with corresponding noncancerous tissues, and gastric cancer patients with high NTN4 expression had favorable survival, suggesting that NTN4 exerts a protective function against cancer progression.

In conclusion, we have clearly demonstrated that CD204^+^ M2-like TAMs are able to suppress NTN4 expression by upregulating miR-210 in gastric cancer cells, thereby leading to increased cell migration. CD204 + M2-like TAMs induce miR-210 expression mainly through TNF-α that activates the NF-κB/HIF-1α pathway. This study provides new insight into the molecular function of miR-210 in the communication between gastric tumor cells and M2-like TAMs, which represents a viable therapeutic target.

## Materials and methods

### Cell culture

The human gastric cancer cell lines, AGS and NCI-N87 were purchased from Food Industry Research and Development Institute (Hsinchu, Taiwan). The human gastric cancer cell lines, HR and MKN45, and the duodenal cancer cell line AZ521 were provided by Prof. Chia-Jui Yen (Department of Medical Oncology, National Cheng Kung University Hospital, Tainan, Taiwan). The human leukemic monocyte lymphoma cell line U937 was a kind gift from Prof. Ming-Derg Lai (Department of Biochemistry and Molecular Biology, College of Medicine, National Cheng Kung University, Tainan, Taiwan). All the cell lines were maintained in RPMI-1640 medium (HyClone) supplemented with 10% fetal bovine serum (HyClone), 15 mM HEPES (Biological Industries), 2 mM L-glutamine (Caisson), and 1x antibiotic-antimycotic solution (1,000 units/L penicillin, 2.5 µg/L amphotericin B, and 1000 µg/L streptomycin) (Caisson) and were cultured at 37 °C in a humidified atmosphere containing 5% CO2 and 95% air.

#### ***In vitro*** coculture assay

Transwell inserts with 0.4 μm pore size (BD Bioscience) were used for coculture assays. AGS, NCI-N87, AZ521, HR, or MKN45 cells (1 × 10^5^/well) were seeded in the upper chamber inserts to attach overnight respectively. The next day, 1 × 10^5^ U937 cells were placed in the lower chamber. The coculture was incubated for 72 h.

### Total protein extraction

Cells were collected and lysed in RIPA lysis buffer (Millipore) supplemented with protease inhibitor cocktail (Roche). The samples were incubated for 30 min at 4 °C with constant agitation followed by centrifugation at 14,000 rpm for 10 min at 4 °C. The supernatants were transferred to new 1.5 mL tubes and stored at -80 °C. Protein concentration was determined by the BCA protein assay (Pierce).

### Isolation of nuclear and cytoplasmic proteins

Cells were harvested and washed once with PBS, followed by resuspension in low-salt permeabilization buffer (10 mM HEPES, pH 7.4, 10 mM KCl, 50 µg/mL digitonin) containing protease inhibitors for 30 min at 4 °C. After centrifugation at 3,000 g for 5 min at 4 °C, the supernatants were collected and saved as cytosolic extracts. The pallets were washed with low-salt permeabilization buffer and then extracted with RIPA lysis buffer, followed by centrifugation at 14,000 rpm for 10 min. The supernatants were saved as nuclear protein. Protein concentration was measured by the BCA protein assay (Pierce).

### Western blot analysis

Mixtures containing 30 µg total protein and 6× loading dye were heated at 95 °C for 5 min before being loaded onto SDS-polyacrylamide gels to separate proteins. The proteins were transferred to PVDF membrane (Millipore). The membrane was blocked with 5% milk diluted in 0.05% TBST for 1 h at room temperature followed by incubation with primary antibodies diluted in 0.05% TBST buffer overnight at 4 °C. After washing three times for 10 min each in 0.05% TBST, the membrane was incubated with HRP-conjugated secondary antibodies diluted in 0.05% TBST buffer for 1 h at room temperature. The blots were developed using Immobilon Western Chemiluminescent HRP Substrate (Millipore) according to the manufacturer’s instruction and were captured by the Biospectrum Imaging System (UVP Ltd.). All antibodies used in this study were listed in Table S1.

### RNA extraction

Total RNA of cell lines and gastric cancer specimens was extracted using TRIzol reagent (Invitrogen) and incubated at room temperature for 10 min. After addition of 200 µL chloroform, the samples were mixed gently by hand and incubated at room temperature for 5 min, followed by centrifugation at 12,000 g at 4 °C for 15 min. The aqueous phase supernatant was carefully transferred to a new tube and mixed gently with 700 µL isopropyl alcohol. After incubation on ice for 2 h, the samples were centrifuged at 12,000 g at 4 °C for 10 min. The supernatant was discarded, and the RNA pellet was washed with 75% ethanol and was air-dried at room temperature for few minutes. The dry pellet was resuspended in nuclease-free water and the RNA concentration was determined by the ratio of spectrophotometric absorbance of the sample at 260 nm to that of 280 nm.

### Reverse transcription

One µg total RNA was reverse transcribed for each cDNA. Firstly, each RNA sample was mixed with 1 µL oligo-dT primer (0.5 µg/µL; Promega) and heated at 95 °C for 5 min to disrupt the secondary structure of mRNA. The samples were immediately cooled on ice and mixed with 1 µL dNTP mix (10 mM; Promega), 0.5 µL recombinant RNasin ribonuclease inhibitor (40 units/µL; Promega), 4 µL M-MLV 5× reaction buffer (Promega), 1 µL M-MLV reverse transcriptase (200 units/µL; Promega) and nuclease-free water to bring the final volume to 20 µL. The reaction mixtures were incubated in a thermocycler (Bio-Rad) at 42 °C for 60 min and 70 °C for 15 min and were held at 4 °C. The product was stored at -20 °C for further use.

### Quantitative real time polymerase chain reaction (qPCR) for mRNAs

One µL cDNA template was added to 9 µL mixed reagent containing 1 µL forward primers (10 µM), 1 µL reverse primers (10 µM), 5 µL GoTaq qPCR master mix (2×; Promega), and 2 µL nuclease-free water in a 96-well plate (Applied Biosystems). The plate was sealed and centrifuged at low speed for 1 min to collect all reaction components at the bottom. The reaction was performed using the StepOne Real-Time PCR System (Applied Biosystems), and the protocol was set as follows: initial denaturation at 95 °C for 5 min, 40 cycles of denaturation at 95 °C for 10 s, annealing at 60 °C for 10 s, and extension at 72 °C for 10 s. During the last cycle, the length of the extension step was increased to 7 min, and finally the temperature was held at 4 °C.

### qPCR assay for mature miRNAs

Each reverse transcription reaction for miRNA detection was carried out using 30 ng of total RNA extracted from cancer cell lines or human tumor specimens. The miRNA qPCR was performed using the TaqMan MicroRNA Reverse Transcription Kit (Applied Biosystems), TaqMan Universal PCR Master Mix (Applied Biosystems), and TaqMan MicroRNA Assay primers for human miR-210 (Applied Biosystems), according the manufacturer’s instructions. Amplification and detection of the qPRC products were performed with the StepOne Real-Time PCR System (Applied Biosystems). The levels of miRNAs were normalized to U44 controls.

### In situ hybridization (ISH)

ISH for miR-210 in deparaffinized human tumor tissues was performed according to a previously published protocol [51]. Tissue sections were fixed in 4% paraformaldehyde for 20 minutes at room temperature and treated with proteinase K (Clontech) for 15 minutes at 37°C. The sections were then incubated with pre-hybridization solution (Biochain) for 4 hours at 60°C followed by hybridization with the probe cocktail containing 10 nM digoxigenin-conjugated probe (Exiqon) in hybridization solution (Biochain) at 60°C overnight. The sequences of the probes containing the dispersed locked nucleic acid modified bases with digoxigenin conjugated to the 5’ end were: miR-210 5’-TCAGCCGCTGTCACACGCACAG-3’. After hybridization, the sections were incubated with blocking solution (Biochain) for 1 h at room temperature and then AP-conjugated antidigoxigenin antibodies (Roche) for 1 h at room temperature. After washing with alkaline phosphatase buffer (Biochain), the probe/target complexes were visualized by chromogen nitroblue tetrazolium and bromochloroindolyl phosphate (Biochain).

### Immunohistochemistry (IHC)

A total of 142 gastric tumor tissue specimens were collected from patients who underwent gastrectomy in National Cheng Kung University Hospital. The study protocol was reviewed and approved by the Institutional Review Board of National Cheng Kung University Hospital. The slides of paraffin-embedded tissue sections were put into xylene for 10 min three times to remove paraffin, followed by 100% ethanol for 5 min twice, 95% ethanol for 5 min twice, and 75% ethanol for 5 min twice for rehydration. After PBS wash, slides were incubated in sodium citrate buffer (10 mM, pH 6.0) and heated by autoclave for 10 min at 121 °C for antigen retrival. The endogenous peroxidase activity was quenched by incubation in methanol containing 3% hydrogen peroxide for 30 min. The sections were incubated with blocking buffer (Thermo) at room temperature for 30 min and then incubated with primary antibodies diluted in antibody diluents (DAKO) overnight at 4 °C. After washing three times with PBS, the slides were incubated with biotinylated secondary antibodies (DAKO) for 30 min at room temperature. The immunoreaction products were visualized using the DAB chromogen system (DAKO), and the slides were counterstained with hematoxylin.

### Transient transfection with oligonucleotides

Cells at 70% confluence in 6-well plates were transfected with miRNA mimics or miRNA hairpin inhibitors (100 pmol; Ambio) using Lipofectamine 2000 transfection reagent (Invitrogen) according to the manufacturer’s instruction. Cells were harvested 48 h after transfection.

### Construction of the 3’-UTR-luciferase plasmids and reporter assay

The netrin-4 (NTN4) 3’UTR possessing the predicted miR-210 target site was amplified from human genomic DNA by PCR using the primers listed in Table S2. The *NTN4* 3’UTR was then inserted downstream of the luciferase in the pMIR-REPORT luciferase vector which was a kind gift from Dr. Tse-Ming Hong (National Cheng Kung University, Taiwan). Site-directed mutagenesis of the miR-210 target site within the *NTN4* 3’UTR was carried out using the primers *NTN4*-UTR-mut-F 5’-TCTGTACCTGTATGTGACCACTATACATAGTTTCTTTTGTAC-3’ and *NTN4*-UTR-mut-R 5’-AGTGGTGGTCACATACAGGTACAGAAGATGAATAATAATGAAA-3’. For reporter assays, cells were transiently transfected with the reporter plasmids and the artificial mimic of miR-210 (Applied Biosystems) using Lipofectamine 2000 (Invitrogen). To correct for transfection efficiency, the Renilla luciferase encoding plasmids were cotransfected into cells with the reporter plasmids. Forty-eight hours after transfection, luciferase activity was measured by chemoluminescence assay using the Dual-Luciferase Reporter Assay system (Promega).

### Migration assay

In vitro cell migration assays were performed using Transwell chambers (24-well, 8-µm pore size; BD Biosciences). The lower chamber was filled with 600 µL of growth medium containing 20 µg/mL fibronectin (Sigma). 1 × 10^5^ cells in 100 µL of serum free medium were seeded in the upper chamber and incubated at 37 °C for 8 h. Cells in the upper side of the membrane were removed gently with a cotton swab and rinsed. Cells that migrated through the membrane and attached to the bottom of the membrane were fixed in methanol for 10 min at room temperature, followed by staining with hematoxylin (Merck).

### Wound healing assay

AGS cells were cultured alone or cocultured with U937 cells for 72 h, and were collected for would healing assay. The collected AGS cells were grown in the ibidi Culture-Insert 2 well (Thermo). When both wells were filled with adherent cells, a cell-free wound was generated after removing the insert. Cell migration into the wound area was monitored and photographed at 0 and 16 h using an optical microscope.

### Tumor formation in BALB/c nude mice

CAnN.Cg-Foxn1nu/Crl (BALB/c Nude) mice were purchased from the BioLASCO Taiwan (Bltw). The mixtures of AGS cells and U937 cells (1:1; 1 × 10^6^) were subcutaneously injected into the mice (age of 6–8 weeks old) to form tumors. After 4 weeks of tumor growth, tumors from the group of mice with ascites (n = 5 at least) and the group of mice without ascites (n = 5 at least) were collected for further histological examination. All procedures were approved by the Institutional Animal Care and Use Committee of NCKU.

### Statistical analysis

Statistical analysis of the data performed using the Student’s t-test and ANOVA. Values expressed as means ± SEM of triplicate determinations. Overall survival curves were plotted using the Kaplan-Meier method and the differences were calculated using log-rank test. *P* value less than 0.05 was considered statistically significant.

### Electronic supplementary material

Below is the link to the electronic supplementary material.

Supplementary Material 1

Supplementary Material 2

## Data Availability

The authors declare that all data supporting the findings of this study are available within the paper and its supplemental information files.

## Abbreviations

EMT: Epithelial-mesenchymal transition
LPS: Lipopolysaccharides
IHC: Immunohistochemistry
iNOS: inducible nitric oxide synthase
ISH: In situ hybridization
miRNA: microRNA
NTN4: Netrin-4
qPCR: Quantitative real time polymerase chain reaction
TAM: Tumor-associated macrophage
Th2: T helper type 2
TME: Tumor microenvironment
3’ UTR: 3’ Untranslated region
