# Supplementary material for: CD204-positive M2-like tumor-associated macrophages increase migration of gastric cancer cells by upregulating miR-210 to reduce NTN4 expression
Source: Cancer Immunol Immunother. 2024 Jan 4;73(1):1. doi: 10.1007/s00262-023-03601-5 (PMC10766795; doi:10.1007/s00262-023-03601-5)
Supplement: Supplementary file 1 — Supplementary Material 1 [file 262_2023_3601_MOESM1_ESM.docx]

**Supplementary data**

**Tumor-associated macrophages increase migration of gastric cancer cells by upregulating miR-210 to reduce NTN4 expression**

Chin-Wong Chen^1^*, Hao-Chen Wang^2,3^*, I-Min Tsai^2^, I-Shu Chen^4^, Chang-Jung Chen^2^, Yu-Lun Chen^2,5^, Ya-Chin Hou^5^, and Yan-Shen Shan^2,5^

^1^Department of Surgery, Kaohsiung Veterans General Hospital Yong-Kang Branch, Tainan, Taiwan.

^2^Institute of Clinical Medicine, College of Medicine, National Cheng Kung University, Tainan, Taiwan.

^3^Medical Imaging Center, Innovation Headquarter, National Cheng Kung University; Tainan, Taiwan.

^4^Department of Surgery, Kaohsiung Veterans General Hospital, Kaohsiung, Taiwan.

^5^Department of Surgery, National Cheng Kung University Hospital, College of Medicine, National Cheng Kung University, Tainan, Taiwan.

* These authors contributed equally to this work.

**Correspondence:**

Yen-Shen Shan M.D., PhD

Distinguished Professor

Division of General Surgery, Department of Surgery, National Cheng Kung University Hospital, College of Medicine, National Cheng Kung University

Institute of Clinical Medicine, College of Medicine, National Cheng Kung University

138, Sheng-Li Road, Tainan 70428, Taiwan

E-mail: [ysshan@mail.ncku.edu.tw](mailto:ysshan@mail.ncku.edu.tw)


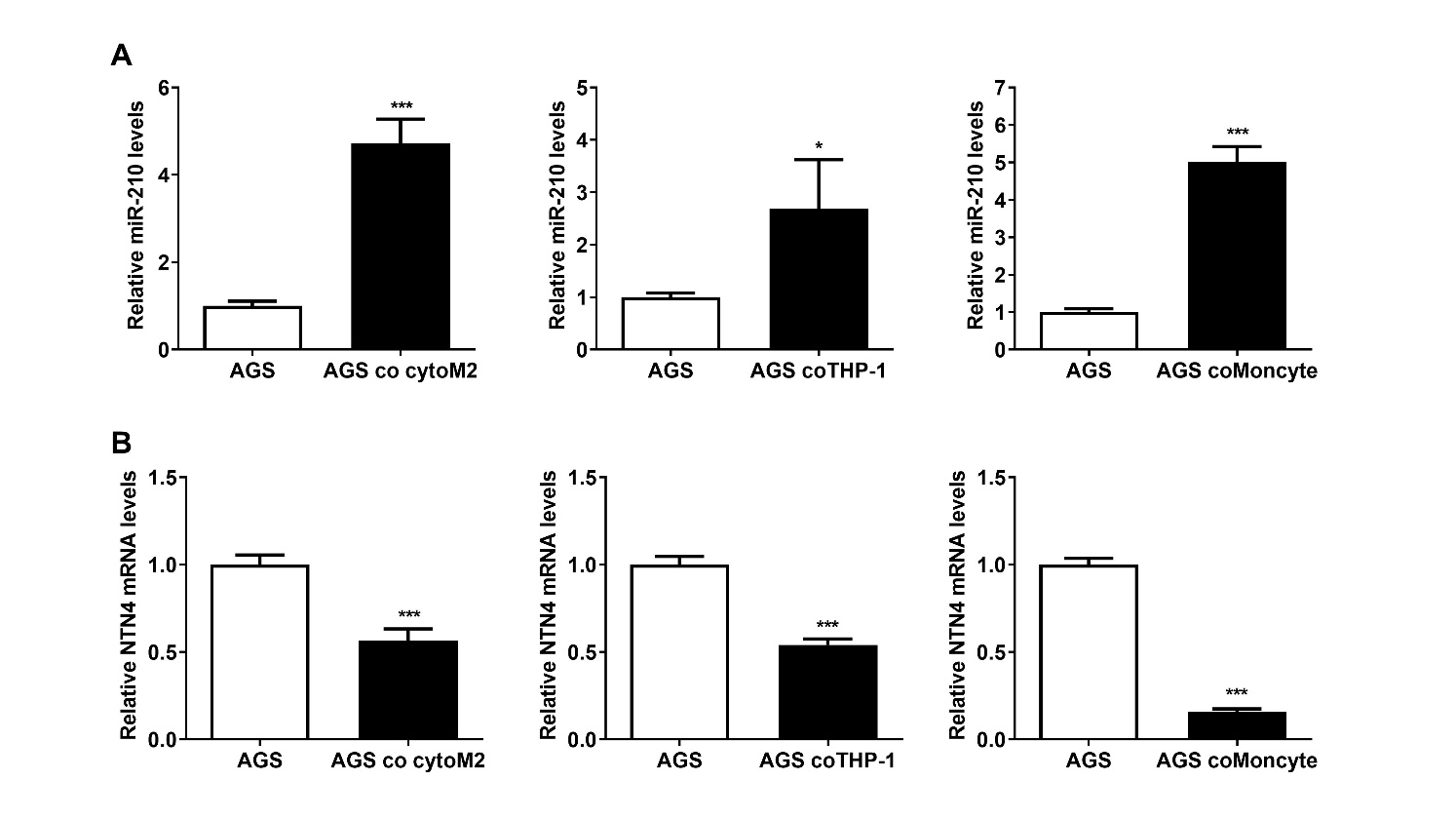


**Supplementary Figure S1. Coculture with cytokine-induced M2 macrophages, THP-1 cells, or monocytes upregulates miR-210 but downregulates NTN4 mRNA expression in AGS cells.** AGS cells were cocultured with M2 macrophages induced by both IL-4 (20 ng/mL) and IL-10 (20 ng/mL) from U937 cells, monocytic THP-1 cells, or CD14^+^ monocytes isolated from healthy human peripheral blood for 72 hours. **(A)** miR-210 and **(B)** NTN4 mRNA expression in AGS cells after monoculture or coculture were measured by qPCR. The bar graph depicts the relative miR-210 and NTN4 mRNA expression. * *P* < 0.05; *** *P* < 0.001 versus monoculture, Student’s t-test.

**
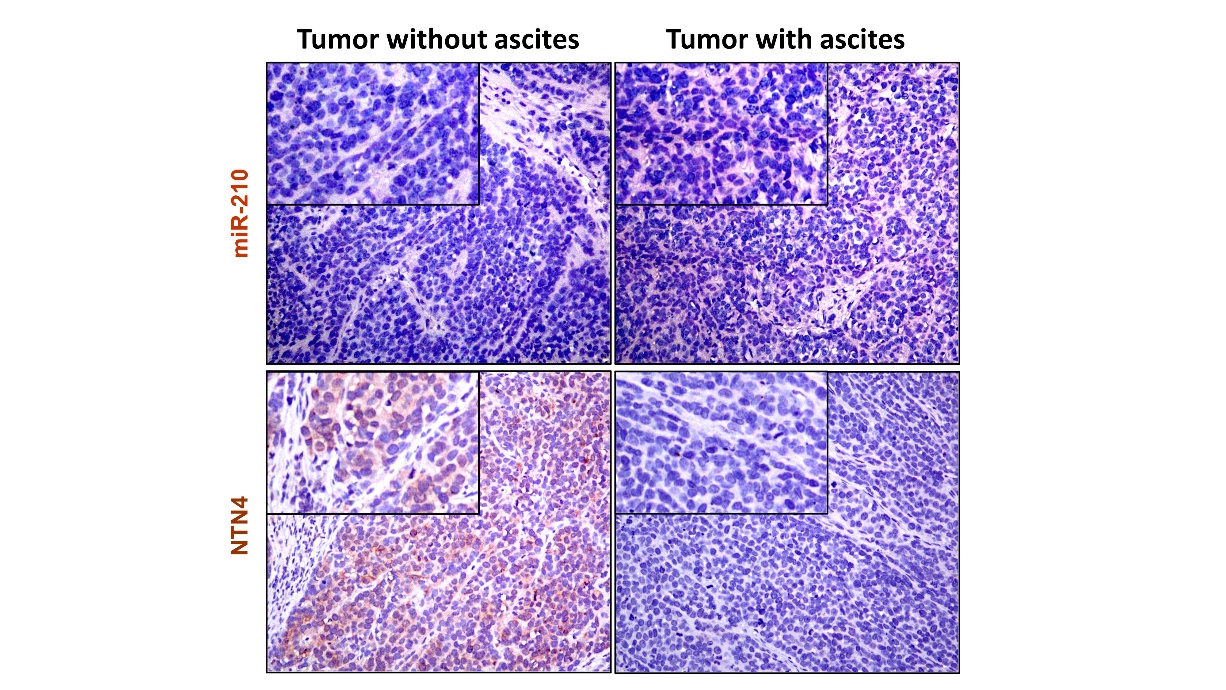
**

**Supplementary Figure S2. miR-210 is inversely correlated to NTN4 protein expression in SCM-1 tumors of mice with ascites or without ascites.** SCM-1 cells were subcutaneously injected into BALB/c nude mice to form tumors. After 4 weeks, tumors from mice with ascites or without ascites were collected. Expression of miR-210 and NTN4 protein was detected by ISH and IHC, respectively.
