# Supplementary material for: CD204-positive M2-like tumor-associated macrophages increase migration of gastric cancer cells by upregulating miR-210 to reduce NTN4 expression
Source: Cancer Immunol Immunother. 2024 Jan 4;73(1):1. doi: 10.1007/s00262-023-03601-5 (PMC10766795; doi:10.1007/s00262-023-03601-5)
Supplement: Supplementary file 2 — Supplementary Material 2 [file 262_2023_3601_MOESM2_ESM.docx]

| **Table S1.** List of antibodies used in this study | | | | |
| --- | --- | --- | --- | --- |
| **Primary Antibody** | **Application** | **Dilution** | **Company** | **Catalog No.** |
| Goat anti-macrophage scavenger receptor 1 (CD204) | IHC | 1:500 | GeneTex | GTX74366 |
| Mouse anti-HIF-1α | WB | 1:1000 | NOVUS | NB100-123 |
| Rabbit anti-Lamin A/C | WB | 1:10000 | abcam | ab108922 |
| Mouse anti-Netrin-4 | WB | 1:500 | Sigma | N3912 |
| Mouse anti-Netrin-4 | IHC | 1:50 | Santa Cruz | sc-365280 |
| Rabbit anti-NF-κB p65 | WB | 1:10000 | abcam | ab32536 |
| Rabbit anti-Occludin | WB | 1:250 | abcam | ab31721 |
| Rabbit anti-α-SMA | WB | 1:400 | abcam | ab5694 |
| Rabbit anti-Vimentin | WB | 1:1000 | GeneTex | GTX100619 |
| Mouse anti-β-actin | WB | 1:5000 | Thermo | MA5-15739 |
| **Secondary Antibody** | **Application** | **Dilution** | **Company** | **Catalog No.** |
| AP-conjugated sheep anti-Digoxigenin | ISH | 1:200 | Roche | 11093274910 |
| LSAB+ System-HRP anti-goat IgG | IHC | 1:1 | DAKO | K0690 |
| HRP-conjugated goat anti-mouse IgG | WB | 1:5000 | Chemicon | AP124P |
| HRP-conjugated goat anti-rabbit IgG | WB | 1:5000 | abcam | ab6721 |
| HRP conjugated rabbit anti goat IgG | WB | 1:5000 | GeneTex | GTX26741 |

| **Table S2.** List of primers used in this study | | |
| --- | --- | --- |
| **Target** | **Application** | **5’→ 3’ Sequence** |
| NTN4 | qPCR | F: AGACTGGTATCATGAAGTTCC |
|  |  | R: TTACCTGAGTGTAGAAGTGC |
| GAPDH | qPCR | F: GGGGCTCTCCAGAACATCAT |
|  |  | R: GTCGTTGAGGGCAATGCCAG |
| NTN4 3’UTR | Reporter construction | F: CCTGAACATTGAAACAGTTTTCC |
|  |  | R: TCCACAGGACTTCAGAGAAAAA |
| NTN4-UTR-mut | Site-directed mutagenesis | F:TCTGTA CCTGTATGTGACCACTATACATAGTTTCTTTTGTAC |
|  |  | R:AGTGGTGGTCACATACAGGTACAGAAGATGAATAATAATGAAA |
| F: forward primer, R: reverse primer | | |

| **Table S3.** 105 potential target genes of miR-210 identified by mRNA array and miRNA database | | | | | |
| --- | --- | --- | --- | --- | --- |
| **Ranking** | **Gene name** | **Fold change** | **Ranking** | **Gene name** | **Fold change** |
| 1 | *STEAP1B* | -3.958 | 54 | *PARP14* | -1.266 |
| 2 | *PHLDB2* | -3.352 | 55 | *EFNB2* | -1.259 |
| 3 | *SH3BGR* | -2.888 | 56 | *ALPK1* | -1.249 |
| 4 | *SLC7A11* | -2.851 | 57 | *NCOA5* | -1.249 |
| 5 | *SERPINA3* | -2.711 | 58 | *DST* | -1.228 |
| 6 | *SERPINB8* | -2.655 | 59 | *NDE1* | -1.224 |
| 7 | *NTN4* | -2.608 | 60 | *BACE1* | -1.208 |
| 8 | *B3GAT1* | -2.532 | 61 | *CCDC66* | -1.205 |
| 9 | *CEBPB* | -2.487 | 62 | *FBXL17* | -1.205 |
| 10 | *CD22* | -2.413 | 63 | *MYH9* | -1.203 |
| 11 | *ARRDC4* | -2.305 | 64 | *PADI1* | -1.187 |
| 12 | *IL8RA* | -2.163 | 65 | *TBC1D2B* | -1.178 |
| 13 | *SH2B3* | -1.969 | 66 | *ABHD2* | -1.172 |
| 14 | *KRT23* | -1.967 | 67 | *GPR87* | -1.166 |
| 15 | *OGT* | -1.958 | 68 | *CC2D1A* | -1.164 |
| 16 | *NUPR1* | -1.894 | 69 | *FAM82B* | -1.163 |
| 17 | *ANKRD1* | -1.870 | 70 | *FAM102A* | -1.161 |
| 18 | *SLC1A4* | -1.844 | 71 | *INHBB* | -1.157 |
| 19 | *TMSB4X* | -1.844 | 72 | *HIVEP2* | -1.156 |
| 20 | *WARS* | -1.835 | 73 | *DUSP12* | -1.148 |
| 21 | *ENOX2* | -1.715 | 74 | *UBQLN1* | -1.142 |
| 22 | *CLDN1* | -1.694 | 75 | *LRRC8D* | -1.126 |
| 23 | *ZNF697* | -1.676 | 76 | *ALKBH3* | -1.124 |
| 24 | *SLC8A1* | -1.639 | 77 | *SH3KBP1* | -1.119 |
| 25 | *PREPL* | -1.637 | 78 | *CD59* | -1.114 |
| 26 | *C9ORF150* | -1.627 | 79 | *CDKN1A* | -1.111 |
| 27 | *TREML2* | -1.603 | 80 | *C14ORF145* | -1.107 |
| 28 | *ERAP1* | -1.598 | 81 | *EZR* | -1.105 |
| 29 | *WDR5B* | -1.576 | 82 | *SRPRB* | -1.099 |
| 30 | *BAMBI* | -1.571 | 83 | *MBD1* | -1.098 |
| 31 | *FOSL1* | -1.566 | 84 | *FAS* | -1.092 |
| 32 | *ODZ3* | -1.544 | 85 | *MFSD6* | -1.092 |
| 33 | *NHSL1* | -1.523 | 86 | *CTTN* | -1.091 |
| 34 | *CD55* | -1.496 | 87 | *HBS1L* | -1.089 |
| 35 | *ISCU* | -1.490 | 88 | *RSU1* | -1.082 |
| 36 | *KLF10* | -1.439 | 89 | *GPAM* | -1.075 |
| 37 | *ICA1L* | -1.436 | 90 | *ONECUT2* | -1.072 |
| 38 | *IFI44L* | -1.436 | 91 | *SEPHS2* | -1.072 |
| 39 | *ABCA10* | -1.435 | 92 | *TCHP* | -1.070 |
| 40 | *BRUNOL6* | -1.410 | 93 | *SF3B4* | -1.069 |
| 41 | *SLC43A1* | -1.376 | 94 | *VPS13B* | -1.066 |
| 42 | *POTEE* | -1.362 | 95 | *JMY* | -1.059 |
| 43 | *ZBED2* | -1.355 | 96 | *SP140L* | -1.059 |
| 44 | *PPHLN1* | -1.342 | 97 | *MITF* | -1.055 |
| 45 | *ZNF397* | -1.341 | 98 | *CCDC88C* | -1.052 |
| 46 | *SERP1* | -1.324 | 99 | *EPHA2* | -1.038 |
| 47 | *SLC38A1* | -1.322 | 100 | *PKD1* | -1.037 |
| 48 | *FEM1A* | -1.299 | 101 | *RABGAP1L* | -1.032 |
| 49 | *XPO5* | -1.296 | 102 | *HSPBAP1* | -1.028 |
| 50 | *KLF2* | -1.290 | 103 | *FXR2* | -1.022 |
| 51 | *LRRC8A* | -1.289 | 104 | *COX19* | -1.014 |
| 52 | *STK24* | -1.280 | 105 | *ESRP1* | -1.010 |
| 53 | *CSPP1* | -1.275 |  |  |  |
